# Biomechanics Analysis in Tissue Engineering

**DOI:** 10.3390/bioengineering13060703

**Published:** 2026-06-19

**Authors:** Rui B. Ruben, Marta Carvalho, Olfa Trabelsi

**Affiliations:** 1 CDRSP, Polytechnic University of Leiria, 2430-028 Marinha Grande, Portugal; 2 UNIDEMI, Department of Mechanical and Industrial Engineering, NOVA School of Science and Technology, Universidade NOVA de Lisboa, 2829-516 Caparica, Portugal; mip.carvalho@fct.unl.pt; 3 Laboratório Associado de Sistemas Inteligentes, LASI, 4800-058 Guimarães, Portugal; 4 Université de Technologie de Compiègne, Biomechanics and Bioengineering UMR CNRS 7338, Centre de Recherche Royallieu, CS 60 319, 60 203 Compiègne Cedex, France; olfa.trabelsi@utc.fr

## 1. Introduction

Tissue engineering lies at the crossroads of biology, medicine, and engineering, playing a crucial role in developing advanced treatments for various pathologies. Successful tissue regeneration hinges on understanding how biological systems respond to their environments, from the musculoskeletal to the respiratory systems.

This field involves analyzing processes from individual cell behavior to whole tissue integration. For instance, biological responses to medical devices, such as stent implantation, are dynamic processes intrinsically linked to the mechanical and biological stimuli acting on the host tissue. Integrating continuum mechanics into tissue engineering has thus become a central framework for predicting outcomes in both lab and clinical settings [1,2].

Furthermore, methodologies used to analyze these systems often draw from broader engineering fields. Robust numerical tools traditionally central to macro-scale injury biomechanics [3] are now being seamlessly translated into tissue engineering. This cross-domain translation relies on the understanding that key computational principles, like non-linear finite element analysis and optimization amid material variability, are scale-agnostic. Whether predicting human body responses to high-energy impacts or tracheal implant interactions with host tissue, the core engineering challenges remain the same. For instance, employing Design of Experiments for surgical decision-making in tracheal implants [4], or utilizing computational mechanics to assist in the customized design of ankle-foot orthoses [5], provides an essential framework for predicting tissue failure and designing protective or regenerative solutions. Addressing this complexity requires the convergence of experimental data with robust computational methodologies. This Special Issue captures these advancements, focusing on experimental and computational methodologies that push the boundaries of the field.

## 2. Overview of the Contributions

The eight articles published in this collection reflect the multidisciplinary nature of contemporary bioengineering, addressing core areas such as scaffold design, soft tissue characterization, and advanced numerical simulations.

Regarding bone tissue engineering and orthopedic stability, Sousa et al. [6] explore the use of Fused Deposition Modeling for personalized bio-scaffolds, highlighting how 3D printing parameters and material selection are crucial for achieving the necessary porosity and mechanical integrity. A complementary perspective is provided by Söhling et al. [7], who offer a rigorous comparison of parameters for measuring bone healing, demonstrating that histological bone fraction correlates significantly with bending stiffness. In clinical interventions, Neto et al. [8] utilize non-linear finite element analysis to investigate the stability of femoral osteotomies, emphasizing that dual-plate configurations and specific allograft fastening techniques significantly enhance system stability.

Soft tissue characterization and clinical biomechanics also represent a significant portion of this issue. Kriener et al. [9] investigate the human abdominal wall, revealing that its tensile properties are not simply additive across its layers—a crucial finding for surgeons and engineers. In neonatal care, Singh et al. [10] address a critical clinical gap by providing experimental data on the forces required for neonatal brachial plexus avulsion using a piglet model. Furthermore, the viscoelastic nature of internal organs is explored by Mishra and Cleveland [11] in the porcine kidney, demonstrating that the semi-fractional Kelvin–Voigt model provides a superior fit for describing the organ’s rheological behavior.

Advanced modeling and biomimetic materials complete the collection, showcasing new computational techniques essential for the future of in silico medicine. McLean et al. [12] present an innovative application of Dynamic Mode Decomposition to analyze the modal properties of the human brain under impact, offering a deeper understanding of injury mechanisms beyond simple force-acceleration criteria. Finally, the quest for accurate tissue mimics is addressed by Mishra and Cleveland [13], evaluating agarose gels as surrogates for the heart, kidney, and liver. By using a springpot model, the authors successfully identified specific agarose concentrations that match the mechanical signature of these organs, paving the way for more realistic surgical simulators.

## 3. Knowledge Gaps and Future Directions

While computational modeling serves as a crucial intersection where these diverse disciplines meet [14], significant knowledge gaps remain. Notably, there is a lack of validated, cross-platform biomechanical scoring systems that link experimental outcomes with in silico results.

The transition to in silico tissue engineering signifies the next frontier, moving away from traditional trial-and-error methods to a digital-first approach [15]. Importantly, this digital revolution depends on a symbiotic framework where high-resolution, non-destructive experimental data, such as microstructural fiber tracking and tissue clearing, directly informs and validates these virtual environments. To bridge this gap between the physical and digital domains, future research should focus on:

- Non-Destructive Optical Characterization: Implementing advanced imaging techniques like Optical Coherence Tomography (OCT) to track soft tissue microstructural evolution in real time [16,17].
- Patient-Specific Digital Twins: Integrating clinical multi-scale imaging with computational predictive models to transition towards robust, personalized in silico environments [15].
- Bio-intelligent Scaffolds: Advancing from static constructs to sensor-embedded biomaterials that adapt dynamically to the host’s mechanical environment and accelerate regeneration [6].
- Standardized Mechanobiological Metrics: Establishing universal parameters and comprehensive scoring systems that directly correlate structural mechanical integrity with biological tissue maturity [7].

## 4. Conclusions

This Special Issue emphasizes that the future of tissue engineering depends on integrated biomechanical analysis. The key message is that successful tissue regeneration requires a unified approach to structural mechanics and biological growth, leveraging their dynamic feedback loop. By merging computational predictions with high-fidelity, non-destructive experimental validation, the field is making strides toward transforming static biomaterials into functional, patient-specific living tissues. As Guest Editors, we hope this collection inspires the next generation of bioengineering innovations and thank the authors and reviewers for their dedication to this multidisciplinary endeavor.
